# Harnessing synaptic plasticity for sustained motor improvement in Parkinson’s disease

**DOI:** 10.4103/NRR.NRR-D-25-00636

**Published:** 2025-09-29

**Authors:** Srdjan Sumarac, Nader Pouratian, Aryn H. Gittis, Luka Milosevic

**Affiliations:** Institute of Biomedical Engineering, University of Toronto, Toronto, ON, Canada; Krembil Brain Institute, University Health Network, Toronto, ON, Canada; Department of Neurological Surgery, UT Southwestern Medical Center, Dallas, TX, USA; Department of Biological Sciences & Neuroscience Institute, Carnegie Mellon University, Pittsburgh, PA, USA

**Figure 1:**
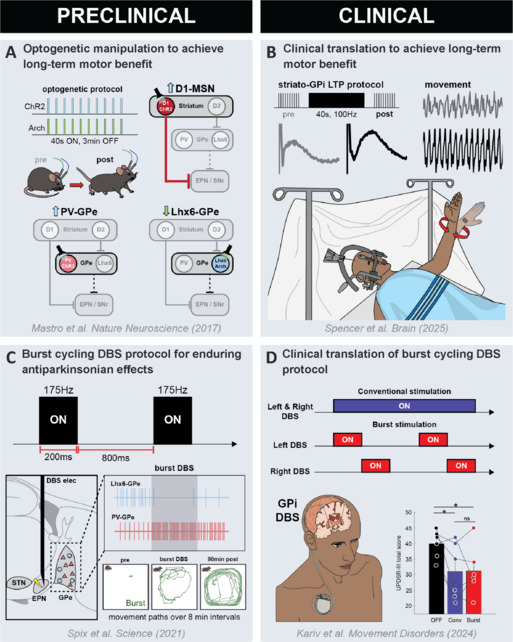
Translational insights from preclinical studies to clinical DBS applications. (A) Optogenetic manipulations (40-second ON/3-minute OFF) of basal ganglia circuits from Mastro et al. (2017) demonstrated sustained motor improvements in dopamine-depleted rodents through activation of D1-MSN and PV-GPe neurons and suppression of Lhx6-GPe neurons. (B) Clinical translation of striato-GPi LTP-like plasticity (Spencer et al., 2025) using 40-second trains of 100 Hz HFS increased striatum-mediated fEP amplitudes and improved motor performance in PwPD. (C) A burst cycling DBS protocol (Spix et al., 2021) using short intermittent stimulation (200-ms bursts of 175 Hz, every 1 second) selectively modulated GPe neuronal subpopulations, restoring movement beyond stimulation cessation. (D) Clinical application (Kariv et al., 2024) of burst DBS (200-ms bursts of 150 Hz, every 1 second) demonstrated equal efficacy to conventional DBS, safely improving motor symptoms as measured by UPDRS-III scores. Arch: Archaerhodopsin-3; ChR2: channelrhodopsin-2; DBS: deep brain stimulation; D1-MSN: D1-receptor-expressing medium spiny neuron; EPN: entopeduncular nucleus; fEP: field-evoked potential; GPe: external segment of the globus pallidus; GPi: internal segment of the globus pallidus; Lhx6-GPe: LIM homeobox 6-expressing external globus pallidus neuron; LTP: long-term potentiation; PV-GPe: parvalbumin-expressing external globus pallidus neuron; PwPD: people with Parkinson’s disease; STN: subthalamic nucleus; UPDRS-III: Unified Parkinson’s Disease Rating Scale, motor subsection.

Deep brain stimulation (DBS) is an established therapeutic intervention for people with Parkinson’s disease (PwPD) and is increasingly being utilized for other neurological disorders. Although effective in alleviating motor symptoms and reducing medication requirements, DBS has undergone minimal conceptual evolution and still relies on continuous high-frequency electrical stimulation. In Parkinson’s disease (PD), this persistent stimulation may cause adverse effects, including dysarthria, stimulation-induced dyskinesia, impulsivity, and mood alterations. Additionally, the continuous energy demand of current DBS systems accelerates battery depletion, necessitating more frequent battery charging or battery replacement surgeries, increasing risks, burden, and costs. Basic neuroscience research has long demonstrated that exogenous electrical stimulation can induce persistent changes to synaptic connections, known as long-term plasticity. This raises the question of whether continuous DBS could be replaced by stimulation paradigms leveraging plasticity for therapeutic effects that persist even after stimulation ceases. Such approaches have recently been demonstrated in Parkinsonian rodent models (**Figure 1A** and **C**) and PwPD (**Figure 1B** and **D**). In general, the field still lacks robust bench-to-bedside translation, with limited incorporation of mechanistic insights into clinical DBS protocols. A critical re-evaluation of existing DBS strategies, with an emphasis on harnessing lasting physiologically-informed plastic changes to modulate circuit function, may yield more effective therapeutic strategies that minimize stimulation-related side effects and energy demands to reduce therapeutic burden, risks, and costs.

According to the canonical basal ganglia circuit model, movement is regulated by two primary pathways originating from the striatum. The direct pathway sends inhibitory signals directly to the globus pallidus internus (GPi) and substantia nigra pars reticulata (SNr), facilitating movement by disinhibiting thalamocortical circuits. In contrast, the indirect pathway inhibits the globus pallidus externus (GPe), disinhibiting the subthalamic nucleus (STN), ultimately suppressing movement by enhancing inhibitory output from the basal ganglia nuclei, GPi and SNr. Two distinct populations of medium spiny neurons (MSNs) in the striatum underlie these pathways: those expressing excitatory G-protein coupled dopamine type 1 (D1) receptors primarily govern the direct pathway, while those expressing inhibitory G-protein coupled dopamine type 2 (D2) receptors mediate the indirect pathway. In PD, the loss of dopaminergic cells in the substantia nigra pars compacta leads to underactivity of D1-MSNs and overactivity of D2-MSNs. This imbalance results in overactivity of basal ganglia outputs; thus, over-inhibition of thalamocortical networks, leading to the characteristic hypokinetic symptoms of PD. Supporting this model, recent electrophysiological findings (Sumarac et al., 2024) show that GPi neuronal firing rates are significantly higher in PwPD compared to those with dystonia (which is a hyperkinetic disorder), reinforcing the notion that excessive basal ganglia output may underlie hypokinetic features.

Of relevance, studies in PwPD suggest that targeted neuromodulation strategies can potentiate striatal pathways, leading to prolonged suppression of basal ganglia output nuclei even after stimulation cessation (discussed below). One way to quantify potentiation is by studying changes in the amplitudes of stimulation-evoked field potentials (fEPs), which reflect the summation of transmembrane currents elicited by stimulation pulses, indicating synaptic activations. In the basal ganglia, differentiating inhibitory fEP responses that are of striatal origin from other inhibitory basal ganglia structures, such as the GPe, is essential. To this end, an early animal study in anesthetized cats demonstrated that stimulation of the caudate nucleus (a principal striatal structure) reliably evoked high-latency (4–5 ms post-stimulation), positive-going evoked potentials coupled with neuronal inhibition responses in SNr, which were abolished by picrotoxin (a gamma-aminobutyric acid [GABA] antagonist), directly implicating striatal GABAergic projections in their generation (Precht and Yoshida, 1971). Notably, fEPs with nearly identical time courses and waveform morphologies, which give rise to neuronal inhibitory responses, have been observed in PwPD during local stimulation within both the SNr and GPi, but not STN (Sumarac et al., 2024). Patch-clamp experiments (Steiner et al., 2019) in juvenile rodent STN slices revealed that GPe–STN GABAergic activations exhibit short-latency synaptic responses (2.2–3.7 ms), closely resembling inhibitory fEPs in response to local stimulation in STN of PwPD (Steiner et al., 2024), which are notably distinct from the longer-latency striatal responses. In addition to morphological differences, the inhibitory potentials in STN, assumed to arise from the GPe, remain remarkably sustained under high-frequency stimulation (HFS), whereas striatal inhibitory responses in GPi and SNr progressively weaken due to the limited capacity of striatal neurons to sustain continuous neurotransmitter release (Steiner et al., 2022). Taken together, these differences in evoked potential latency, morphology, and synaptic dynamics enable differentiation between striatum-mediated fEPs in GPi and SNr *versus* GPe-mediated fEPs in STN.

Of importance, it has been demonstrated that HFS can induce long-term potentiation (LTP) -like changes to striatum-mediated fEPs in both the SNr (Milosevic et al., 2019) and the GPi (Sumarac et al., 2024). In these studies, inhibitory striatal responses were evoked by delivering electrical pulses through a dedicated stimulating electrode while an adjacent microelectrode (positioned 600 μm away at the same depth) simultaneously recorded extracellular voltage changes. LTP was quantified by first delivering low-frequency (1 Hz) “test” pulses to establish baseline fEP amplitudes and neuronal silent periods (intervals of suppressed neuronal firing following each pulse). This was followed by trains of 100 Hz HFS, after which the test pulses were repeated to assess HFS-induced changes. Comparisons of baseline and post-HFS measurements revealed significant increases in fEP amplitudes and prolonged silent periods, indicating strengthened inhibitory responses. The strong correlation between these measures demonstrates that fEPs provide a reliable proxy for studying inhibitory activation and plasticity (Milosevic et al., 2019), underscoring the potential for HFS to produce enduring LTP-like changes of striatal inputs to the GPi/SNr and sustained suppression of basal ganglia output. These LTP-like changes are thought to reflect lasting increases in synaptic efficacy that emerge after stimulation, supported by post-tetanic potentiation-driven rebound in GABA release and/or intracellular signaling cascades that enhance both presynaptic release mechanisms and postsynaptic GABAA receptor expression and function.

Given that both of the inhibitory projections from striatum-GPi and GPe-STN are functionally underactive in PD, recent work (Spencer et al., 2025) evaluated whether HFS-induced LTP-like plasticity may produce sustained antiparkinsonian effects. During GPi implantation surgery, a 40-second train of HFS (100 Hz) produced a significant increase in striato-GPi evoked potential amplitudes, accompanied by improved motor performance on a pronation-supination task (**Figure 1B**). Moreover, in the clinic, leveraging access to sensing-enabled GPi-DBS, it was found that this protocol delivered through the chronic DBS implant could also produce sustained motor improvements and reduce power of beta frequency oscillations. In contrast, applying the same paradigm to the STN, including a higher-frequency variant (180 Hz), failed to induce LTP-like effects of GPe-mediated fEPs, motor improvements, or lasting reductions in beta activity. Notably, motor performance before *versus* after stimulation cessation in the STN was unchanged, whereas GPi stimulation produced improvements that outlasted active stimulation. While these results suggest that STN-DBS does not induce LTP-like plasticity, its acute effects may arise from short-term network modulation, including upregulation of GPe activity, which may contribute to network desynchronization and rebalancing of the indirect pathway. In contrast, GPi-DBS additionally engages and potentiates direct pathway D1-mediated striatal projections, which may permit an enduring post-stimulation response. These findings in PwPD align with preclinical work (Mastro et al., 2017), which used optogenetic photostimulation in dopamine-depleted mice to test whether activating D1-MSNs or GPe neurons could restore movement (**Figure 1A**). It was found that global GPe photostimulation using channelrhodopsin-2 under the human synapsin-1 promoter increased firing rates yet failed to reduce immobility or bradykinesia. However, activating D1-MSNs, which project to the entopeduncular nucleus (the rodent equivalent of the GPi) and SNr produced immediate and prolonged motor improvements. Interestingly, it was also shown that selective activation of parvalbumin-expressing GPe neurons (PV-GPe) reduced immobility and bradykinesia with effects lasting hours, while inhibition of Lim homeobox 6-expressing GPe neurons (Lhx6-GPe) produced similarly persistent motor recovery. This highlights the functional complexity of GPe circuitry and underscores the need for targeted approaches that achieve durable therapeutic benefits by optimizing DBS protocols.

To this end, in a follow-up study (Spix et al., 2021), the same group investigated whether electrical DBS could replicate the prolonged therapeutic effects of optogenetic neuromodulation in dopamine-depleted mice (**Figure 1C**). Building on prior findings (Mastro et al., 2017), the team examined whether electrical stimulation could differentially modulate PV-GPe and Lhx6-GPe neurons. Using acute brain slices from genetically labeled mice, they recorded extracellular activity during continuous HFS and found that, on average, both populations were upregulated. However, intracellular voltage-clamp recordings revealed that Lhx6-GPe neurons received disproportionately strong but transient inhibitory input, which rapidly reversed over time, whereas PV-GPe neurons remained persistently active. Based on this synaptic asymmetry, the authors hypothesized that brief, intermittent stimulation could selectively modulate these populations by exploiting differences in inhibitory drive. Testing 1-second bursts of 100 Hz stimulation confirmed this, as PV-GPe neurons remained active while Lhx6-GPe neurons were suppressed. To refine selectivity, they systematically varied stimulation parameters, identifying that short, high-frequency bursts (175 Hz, 200 ms, 800 ms interburst intervals) maximized population separation. This finding demonstrated that selective neuromodulation relies on precisely timed bursts rather than continuous stimulation, as prolonged stimulation led to synaptic adaptations that eroded population-specific effects. To assess functional relevance, they applied burst DBS *in vivo*, targeting a region where excitatory STN and inhibitory D1-MSN fibers converged. Remarkably, burst DBS produced motor improvements lasting over four times longer than conventional DBS. Interestingly, however, circuit perturbations confirmed that coactivation of STN and D1-MSN inputs was necessary, as chemogenetic suppression of D1-MSN terminals or pharmacological blockade of STN input abolished the population-specific responses in GPe neurons; however, the behavioral implications of these manipulations were not studied. Of particular relevance to the works of Mastro et al. and Spencer et al., these findings further underscore the potential importance of D1-mediated direct pathway activation and plasticity in sustained DBS efficacy.

Building on this framework, a recent pilot study (Kariv et al., 2024) tested the feasibility of burst DBS in the GPi of PwPD by evaluating its safety, tolerability, and acute therapeutic effects (**Figure 1D**). Six participants with implanted bilateral GPi-DBS systems underwent three conditions: DBS OFF, conventional DBS, and burst DBS, which was delivered as sequential 200-ms bursts at 150 Hz with 800-ms interburst intervals, similar to the burst stimulation paradigm developed by Spix et al. (2021). Both conventional and burst DBS significantly improved UPDRS-III motor scores and proactive inhibition in a stop-signal task compared to DBS OFF, with no significant differences between the two paradigms. However, this study was limited to acute testing (< 2 hours) and did not assess whether burst DBS could produce prolonged therapeutic benefits after stimulation cessation. Nonetheless, these findings confirm the feasibility and tolerability of burst DBS in PwPD and provide clinical validation that a discontinuous stimulation approach with only 20% ON-stimulation time, as originally tested in dopamine-depleted mice, could at least match the efficacy of continuous stimulation, warranting further investigations of long-term efficacy and possibility of effects enduring beyond stimulation cessation.

Further support for plasticity-driven DBS strategies can also be found in observations of prolonged symptom relief following GPi-DBS cessation compared to STN-DBS. In a clinical trial (Follett et al., 2010) comparing STN and GPi stimulation in 299 PwPD, investigators noted that motor symptoms in GPi-DBS recipients remained suppressed for a longer duration after stimulation was turned OFF, whereas those treated with STN-DBS experienced a more immediate return of symptoms. This extended washout period (i.e., the time between stimulation cessation and the re-emergence of motor symptoms) suggests that GPi-DBS may induce more persistent network effects. Historically, this phenomenon was considered clinically irrelevant since patients were expected to keep their stimulators ON continuously. However, if intermittent stimulation emerges as a viable clinical strategy, the capacity of GPi-DBS to sustain benefits beyond stimulation cessation becomes a clear advantage, offering a foundation for protocols that optimize stimulation timing in ways that may mitigate side effects and would substantially preserve battery life. One practical implementation of this approach could involve delivering low frequency diagnostic pulses during periods when clinical stimulation is turned off, allowing evoked potential amplitudes to serve as a real-time measure of synaptic efficacy.

Despite the promising rationale for LTP-inspired DBS, key questions remain regarding the optimal stimulation parameters to maximize sustained benefits. Experimental and clinical studies in people are needed to identify the optimal combination of frequency, pulse width, amplitude, and duty cycle, with a particular focus on maximizing the duration of motor benefits following stimulation. Longitudinal assessments, paired with biomarkers such as local field potentials and fEPs, could help optimize protocols by providing real-time neurofeedback of how stimulation patterns engage plasticity mechanisms over time. A critical objective is to understand how burst DBS sustains LTP-like effects compared to conventional DBS to refine protocols that maximize benefits. Further, combining or sequentially modulating direct and indirect pathways may yield synergistic benefits, providing a more comprehensive strategy for PD. Through these refinements, DBS can move beyond continuous stimulation toward more physiologically-informed and plasticity-driven approaches to restoring circuit function and promoting durable motor recovery in PD.
